# Contralateral Occlusion Increases the Risk of Neurological Complications Associated with Carotid Endarterectomy

**DOI:** 10.1155/2015/942146

**Published:** 2015-01-29

**Authors:** Laura Capoccia, Enrico Sbarigia, Anna Rita Rizzo, Chiara Pranteda, Danilo Menna, Pasqualino Sirignano, Wassim Mansour, Andrea Esposito, Francesco Speziale

**Affiliations:** Vascular and Endovascular Surgery Division, Department of Surgery “Paride Stefanini”, Policlinico Umberto I, “Sapienza” University of Rome, 155 Viale del Policlinico, 00161 Rome, Italy

## Abstract

*Objective*. To report on the incidence and factors associated with the development of perioperative neurological complications following CEA in patients affected by carotid stenosis with contralateral occlusion (CO) and to compare results between those patients and the whole group of patients submitted to CEA at our vascular division from 1997 to 2012.* Methods*. Our nonrandomized prospective experience including 1639 patients consecutively submitted to CEA was retrospectively reviewed. 136 patients presented a CO contralateral to the treated carotid stenosis. Outcomes considered for analysis were perioperative neurological death rates, major and minor stroke rates, and a combined endpoint of all neurological complications.* Results*. CO patients more frequently were male, smokers, younger, and symptomatic (*P* < 0.001), presented with a preoperative brain infarct and associated peripheral arterial disease (*P* < 0.0001), and presented with higher perioperative major stroke rate than patients without CO (4.4% versus 1.2%, resp.,* P* = 0.009). Factors associated with the highest neurological risk in CO patients were age >74 years and preoperative brain infarct (*P* = 0.03). The combination of the abovementioned factors significantly increased complication rates in CO patients submitted to CEA.* Conclusions*. In our experience CO patients were at high risk for postoperative neurological complications particularly when presenting association of advanced age and preoperative brain infarction.

## 1. Introduction

Patients presenting a carotid stenosis and contralateral occlusion (CO) have been historically considered at high risk for carotid endarterectomy (CEA), since results from randomized controlled trials (RCTs) on carotid surgery have reported morbidity and mortality rates significantly higher than in the general population affected by carotid stenosis [1–5]. Hence, that group of patients has been frequently excluded from surgical treatment in some prospective RCTs comparing results in CEA and carotid artery stenting (CAS) in the following years, as in the SAPPHIRE trial. On the other hand, some single-center reports have highlighted a nondissimilar rate of complications in patients presenting with a carotid stenosis with or without contralateral occlusion [6–10]. In those studies perioperative complications are reported in 0.7 to 6.9% of patients presenting with carotid stenosis and contralateral occlusion, thus reaching percentages consistent with international recommendations for CEA postoperative complications in symptomatic patients, but surely exceeding those requested for CEA in asymptomatic ones [11, 12]. However, within the population of patients affected by carotid stenosis and contralateral occlusion, different risk categories could be identified with respect to general medical conditions and involvement of other vascular districts in the atherosclerotic process, as well as with respect to presence of previous brain infarct on neuroimaging. It has to be noted that frequently patients affected by CO have been included in the “high-surgical risk” category mixed with patients presenting general medical conditions at high risk for surgery, thus generating confusion with regard to the real risk addressed by those patients [5, 7, 8, 10].

We retrospectively reviewed our database on carotid endarterectomy to analyse the incidence of postoperative neurological complications in patients affected by carotid stenosis and chronic contralateral occlusion, to analyse factors associated with the development of complications in this group of patients, and to compare their results with those obtained in the group of patients without CO submitted to CEA at our vascular surgery division.

## 2. Methods

From January 1997 to December 2012, 1639 patients underwent primary CEA at our vascular surgery division. Data on demographics, risk factors, preoperative neurological evaluation and imaging, intervention, and 30-day outcomes were prospectively collected in our institutional database. From the database 136 out of 1639 (8.3%) patients presenting a stenosis of internal carotid artery with a contralateral internal carotid occlusion (CO) submitted to carotid endarterectomy in the stenotic side were identified. A carotid stenosis ≥70% in asymptomatic patients or ≥50% (NASCET [1] stenosis evaluation criteria) in symptomatic patients was considered indication for intervention. Patients were considered symptomatic if they presented carotid-related neurological symptoms in the previous six months before operation. Patients submitted to carotid endarterectomy in urgency (within 2 weeks from last neurological symptom ipsilateral to the carotid stenosis or occlusion) were not considered for analysis in the present series because they represent a subset of patients at higher surgical risk.

All patients preoperatively underwent a complete medical examination, assessment of preoperative neurological examination by the neurologist by use of National Institute of Health Stroke Scale (NIHSS) or Rankin Scale evaluation, blood test, electrocardiogram, and carotid duplex ultrasound imaging to assess the degree of stenosis. Brain computed tomography (CT) or magnetic resonance imaging (MRI) was performed in all CO patients and in all control sample patients with symptomatic or ulcerated/irregular carotid plaque detected at duplex ultrasound examination. Before admission, all patients classified as having a carotid occlusion had been previously submitted to at least two different imaging modalities to confirm the obstruction (duplex US, contrast-enhanced supra-aorticvessels CT scans and MRI).

Patients were operated on on cervical block anaesthesia or, alternatively, general anaesthesia whenever a local anaesthesia was not feasible for patient-related causes.

Patients under local cervical anaesthesia were assessed for neurological deficit and pain throughout the entire procedure by hand-grip test.

During operations under general anaesthesia neurological monitoring was performed by transcranial doppler (TCD) whenever possible and adjunctive quantitative electroencephalogram (QEEG). Cerebral protection by Sundt shunting was selectively used when mean velocity in the middle cerebral artery at TCD monitoring decreased to ≤15 cm/sec or a significant alteration in QEEG recording was detected. Patients were maintained under their scheduled ASA (100 mg) or ticlopidine (250 mg) medication and were postoperatively reevaluated by a neurologist. Postoperative neuroimaging was performed only in those patients presenting a postoperative neurological deficit. Follow-up was conducted at our vascular ultrasound laboratory by carotid DUS and clinical examination at 1, 6, and 12 months and then annually. When a postoperative complication had occurred a neurological assessment was scheduled together with additional 3- and 9-month follow-up visits.

### 2.1. Definitions and Statistical Analysis

Outcome measures for analysis were perioperative (30-day) major stroke (assessed as a neurological deficit lasting more than 24 hours and scored as NIHSS ≥ 4) [13], minor stroke (assessed as a neurological deficit lasting more than 24 hours and scored as NIHSS ≤ 3) [13], and stroke-related or neurological death. Water-shed infarction (cerebral border-zone infarctions) and hyperperfusion syndrome (defined as occurrence of severe unilateral headache, acute changes in mental status, vomiting, seizures, focal neurologic deficits, and, ultimately, intracranial hemorrhage) occurrence were also recorded. Neurological morbidity (major + minor stroke) and mortality were analyzed according to clinical preoperative demographics and presentation and intraoperative details and compared between patients with or without carotid occlusion contralateral to the treated carotid stenosis. We initially performed univariate comparisons between outcome measures and preoperative and intraoperative patients variables. Univariate predictors that were significant at *P* < 0.05 were then entered into a multivariate model using logistic regression which was used to generate odds ratios (ORs) and 95% confidence intervals (CIs) for 30-day neurological outcomes, in order to identify a subset of patients at high risk for neurological complications. Statistical significance was set at *P* ≤ 0.05.

## 3. Results

### 3.1. Between Groups Preoperative Factors Analysis

The demographic characteristics of the two groups (CO and control patients) are shown in Table 1. Between groups analysis disclosed that patients with carotid stenosis and contralateral occlusion (CO group) were significantly younger (67.02 ± 7.9 versus 69.72 ± 8.13; *P* < 0.0001), were more frequently males (114 versus 1054; 83.8% versus 68.3%; *P* = 0.001) and more frequently smokers (103 versus 828; 75.7% versus 53.7%; *P* < 0.0001), and presented a significantly higher incidence of peripheral arterial disease (45 versus 248; 33.1% versus 16.1%; *P* < 0.0001) than patients without contralateral occlusion (control group). CO patients referred previous neurological symptoms ipsilateral to the stenosis in 18 cases out of 86, and contralateral, or ipsilateral to the carotid occlusion, in 68 cases.

Analysis of neurological presentation before operation disclosed that CO patients were more frequently symptomatic (86 CO patients presenting preoperative neurological symptoms versus 665 control patients; 63.2% versus 46.4%; *P* < 0.0001) and presented more frequently cerebral infarcts on preoperative brain imaging (65 CO patients versus 379 control patients; 47.8% versus 25.2%; *P* < 0.0001). One vertebral artery was occluded in 9 patients in the CO group and in 25 patients in the control group.

### 3.2. Between Groups Intraoperative Factors Analysis

CO patients were more frequently submitted to CEA under local anaesthesia (93 versus 873; 68.4% versus 58.1%; *P* = 0.02) and had more frequently a shunt implantation after clamping test (40 versus 121; 29.4% versus 8.1%; *P* < 0.0001). Shunt use rate was not statistically different in patients submitted to CEA under local or general anaesthesia in both groups. Mean clamping time was 23.3 ± 13.5 versus 22.9 ± 12.5 minutes in CO versus control group (*P* = 0.28).

### 3.3. Between Groups Neurological Outcome Analysis

Perioperative (30-day) complications analysis disclosed no significant difference in neurological mortality rates between the two groups (*P* = 0.28). Incidence of postoperative minor stroke was not significantly different in the two groups (0.7% in CO group and 0.5% in control group, resp.), while major stroke incidence analysis disclosed a statistically significant difference (4.4% in CO and 1.2% in control group, *P* = 0.009), accounting for higher overall neurological morbidity and overall neurological complication rates in CO group with respect to control group (5.1% versus 1.7%, *P* = 0.01, and 6.6% versus 2.1%, *P* = 0.003, resp.). In CO patients 5 out of 9 perioperative neurological complications were related to technical defects (carotid early thrombosis, 55.5%) while in control cases carotid early thrombosis was responsible for neurological events in 24 out of 26 cases (92.3%). In 4 cases in CO group and 2 cases in control group neurological complications were related to hypoperfusion or reperfusion injuries (44.5% versus 7.7%). No complication occurred in CO patients presenting one vertebral artery occluded and 1 major stroke (carotid thrombosis in the first hours following intervention) occurred in one patient with an occluded vertebral artery in the control group. Results are summarized in Table 2.

### 3.4. Within Group Analysis of Risk Factors for Postoperative Neurological Complications in CO Patients

Analysis of preoperative clinical factors and neurological outcome in CO patients disclosed that neurological complications occurred more frequently in elderly patients (patients with complications versus patients without complications mean age 73.55 ± 4.24 versus 66.55 ± 7.9; 95% CI: 1.76–12.27; *P* < 0.01; patients with complications versus patients without complications and age >74 years *P* = 0.008) and in patients with cerebral infarct on preoperative neuroimaging (*P* = 0.036). Use of shunt did not show to be related to an increased risk of neurological complications (4 patients in the shunt group and 5 patients in the nonshunt group presented overall perioperative neurological complications combined endpoint). All other variables analysed did not show any statistical difference between favourable and unfavourable outcome. Those same factors were not significantly associated with complications in the control group.

### 3.5. Between Group Analysis of Risk Factors for Postoperative Neurological Complications

The abovementioned risk factors (>74 years derived from the intragroup continuous data analysis of age, and preoperative brain damage) were subsequently analyzed for their role in the development of postoperative neurological complications in the whole sample. Odds ratio analysis disclosed that the association of ipsilateral carotid stenosis and contralateral occlusion with bilateral or contralateral preoperative brain ischemic lesion exposed the CEA patient to a higher surgical risk of postoperative neurological complications (OR 8.1, 95% CI 1.72–38.41, *P* = 0.001; and OR 3.2, 95% CI 0.95–10.96, *P* = 0.04, resp., Table 3). Similarly, in CEA patients the association of ipsilateral stenosis and contralateral occlusion with age >74 years increased significantly this risk (OR 11.5, 95% CI 4.08–32.62, *P* < 0.0001). Further, in CO CEA patients the association of age >74 years with ipsi/bilateral or contralateral preoperative brain damage showed an augmented surgical risk of brain lesions (OR 19.9; 95% CI 1.77–224.56, *P* = 0.0006; OR 40.9, 95% CI 5.61–298.05, *P* < 0.0001, resp., Table 4).

Among 11 CO patients presenting both age >74 years and a preoperative brain infarct, 5 (45.5%) presented postoperative neurological complications. Analysis of the causes of postoperative neurological complications in those 5 patients disclosed that one patient suffered from hemorrhagic transformation of a preoperative brain infarct and died; one patient suffered from acute postoperative carotid thrombosis with immediate neurological deterioration and slow recovery in the following months after CEA; one patient with a very small internal carotid artery (maximal external diameter 4 mm) who did not tolerate carotid clamping, in which a 3 × 4 mm Sundt shunt was employed, presented an intraoperative cerebral hypoperfusion with prompt recovery of the postoperative neurological deficit; and two patients presented a postoperative neurological deficit related to the contralateral carotid occlusion (watershed infarct) with a residual small deficit in the postoperative period.

## 4. Discussion

### 4.1. Our Experience

In the present series CEA patients affected by carotid stenosis and contralateral occlusion presented significantly higher perioperative major stroke rate (*P* = 0.009), overall neurological morbidity (*P* = 0.01), and overall neurological complication rates (*P* = 0.003) compared to control group of patients. From our experience the CO sample, as a whole, represents a subset of patients at higher surgical risk for CEA when compared to the general patients affected by carotid stenosis requiring intervention, in accordance with previous reports [1, 3, 8, 14–16]. Nevertheless, when dealing with CO patients, some peculiarities must be taken into account and some ensuing considerations could be made. First, in our series CO patients demographics analysis showed a higher frequency of smoking history, other vascular district involvements in the atherosclerotic process (i.e., peripheral arterial disease), neurological symptoms history, and, of course, presence of previous brain infarction on neuroimaging. Those data are in accordance with previous studies by Julia et al. [17] and Rockman [6]. Moreover, those patients are generally younger when necessitating a carotid revascularization. This seems to define the picture of a population at high risk for any surgical procedure, given the extensive involvement of the blood vessels in the atherosclerotic process [18–23]. This peculiarity is underlined by the presence of a preoperative neurological symptomatology in 64% of patients in our series, since the population analyzed presented more frequently carotid-related symptoms and more frequently a brain infraction on neuroimaging contralateral to the side affected by the stenosis. So the brain in those patients is somewhat more fragile and less prone to tolerate carotid clamping during CEA or also eventual small and clinically silent ischemic brain lesions (eventually accompanied by perilesional oedema) caused by microembolic plaque particles dislodged during CEA. Ultimately, it could be speculated that the brain in CO patients could have less cerebral functional reserve [16]. Sam et al. [24] have recently reported a significant reduction in cerebrovascular reactivity in anterior circulation of the brain hemisphere ipsilateral to a carotid stenoocclusive disease, thus implying that unilateral carotid stenosis affects the vascular reserve of both sides of the brain, so not only the hemisphere ipsilateral to an occlusion, but also the contralateral one. To further support the theory of a hemodynamic impairment in patients with stenosis and contralateral occlusion Oka et al. [25] demonstrated increased cerebral blood flow and cerebrovascular reactivity in both hemispheres 3–6 months after carotid stenosis treatment. In our series in CO patients immediate neurological complications were related to carotid embolism in 5 cases (55%) and to hypo- or reperfusion in 4 cases (45%), thus accounting for a frailer brain in those patients. These data demonstrate relevant differences concerning causes of complications in comparison with the control sample where hypo- or reperfusion were responsible for postoperative complications in 2/36 cases (5.5%), strengthening the assumption that CO patients present with an increased perfusion instability [16].

Such susceptibility of the brain with respect to any type of ischemic insult in those patients is sustained also by the increased need for shunt implantation during CEA in CO, in accordance with previous reports [6, 9, 26]. Some conflicting data are reported in the literature concerning the need for shunt implantation in CO patients: while some authors advocate shunt implantation in all patients with CO [27], others recommend selective shunting [28] or no shunting at all [29, 30]. To add to the matter, a recent study by Goodney et al. [31] showed that shunt use for CO patients during CEA is associated with fewer complications, but only if the surgeon used a shunt as part of his or her routine practice in CEA.

A careful intragroup analysis of CO population has highlighted that some additional preoperative risk factors can enhance the neurological risk in CEA. In our experience presence of brain infarct on preoperative neuroimaging in CO patients decreases the capability of tolerating any kind of ischemic insult, as demonstrated by a 4.4-fold neurological risk increase in this subset of patients (Table 3). From our analysis patients with bilateral or contralateral brain lesions seem at higher risk for neurological complication following carotid revascularization. Unfortunately, no information was available in our database concerning the size of brain ischemic lesions but we can speculate that contralateral damage in our series, namely, ipsilateral to a carotid occlusion, might be quite wide and so it can reinforce the persuasion that those patients' brains are less prone to tolerate any kind of hemodynamic or embolic ischemic insult. On the other hand, an incomplete Willis circle might justify the higher incidence of old brain infarcts and liability to new ischemic events. Moreover, advanced age, defined in our experience by the presence of more than 74 years, further increases the neurological risk by 11.5-fold (Table 4). The elderly seem at higher risk of brain hemodynamic impairment during carotid clamping per se [32]. When advanced age and preoperative brain infarct are combined, the risk of postoperative neurological complications reached prohibitive values in our series (OR 28.6 in CO population, Table 4). Analysis of specific complications developed in 5 out of 11 CO patients presenting both age >74 years and a preoperative brain infarct has confirmed the higher fragility of the brain in those patients. Except for one case of carotid thrombosis, probably related to a technical defect in CEA, in two patients the postoperative neurological deficit was related to a global brain hypoperfusion not clinically evident during CEA and in another one to a hypoperfusion due to a very small carotid artery in which a small shunt had been implanted, thus causing a damage in the hemisphere ipsilateral to the occluded carotid artery. In one last case a preoperative brain lesion underwent a hemorrhagic transformation thus underlining the weakness produced in the blood-brain barrier by a previous damage.

Even if a higher neurological surgical risk in patients affected by carotid stenosis and contralateral occlusion has been reported in numerous series [8, 14, 15], some single-center experiences have derived different conclusions [27, 33]. This is why nowadays no clear indications for carotid treatment are recognized for this group of patients [34].

### 4.2. Data from the Literature

Historical data on CEA patients have shown CO to be a significant risk factor for perioperative development of neurological complications [1–3, 15]. A post hoc analysis of patients enrolled in NASCET [15] and in the Ontario Carotid Endarterectomy Registry [3] reported a significantly higher incidence of 30-day adverse neurological events in patients with CO. More recently two meta-analyses of patients undergoing revascularization when presenting a contralateral carotid occlusion were independently published in 2013 [16, 35].

Antoniou et al. [16] performed a systematic review of electronic information sources to identify studies comparing perioperative and early outcomes of CEA in patients with occluded and patent contralateral carotid arteries. Thirty articles were included for meta-analysis, comprising 27265 patients having undergone 28846 CEAs between 1961 and 2009. Among them 3120 patients presented a carotid occlusion contralateral to the operated side. The authors' last electronic search was run in August 2012 [16]. The incidence of stroke was 3.3% in the occluded contralateral carotid group and 1.9% in the patent contralateral carotid group (OR 1.65; 95% CI 1.30–2.09; *P* < 0.001). No significant heterogeneity among the studies was identified and no statistically significant association between the time of publication of studies and the likelihood of developing perioperative neurological complications was found. Analysis of complications related to routine or selective shunting was not performed given the lack of significant heterogeneity of outcome in the selected studies. The authors concluded that “patients undergoing CEA in the presence of an occluded contralateral carotid artery had increased perioperative and early postoperative risk,” but they also pointed out a wide variety among studies of degree of stenosis, indication for treatment, exclusion of recurrent stenosis, carotid disease diagnostic methods, selection criteria for patients enrolment, examination of the status of collateral vertebrobasilar circulation, combination of results in symptomatic and asymptomatic patients, anesthetic methods, surgical techniques, and shunt use [16].

Faggioli et al. [35] performed a systematic review of papers published up to March 2012 reporting results on patients with carotid stenosis and contralateral occlusion submitted to either CEA or CAS. They included in the analysis 27 papers on CEA and 6 on CAS. The authors reported CO to be considered a significant risk factor for stroke and death in CEA (OR 1.82; 95% CI 1.57–2.11; *P* < 0.00001) but not in CAS (OR 1.22; 95% CI 0.60–2.49; *P* < 0.58) [35]. In CEA studies the authors performed a subgroup analysis reporting separate results for symptomatic patients (OR 2.43; 95% CI 1.07–5.50; *P* = 0.03), asymptomatic patients (OR 1.83; 95% CI 1.25–2.68; *P* = 0.002), and patients included in studies with higher statistical power (OR 1.75; 95% CI 1.44–2.12; *P* < 0.00001), thus concluding that in all analysis CO represents a significant risk factor for adverse neurological events. Furthermore, the analysis performed with respect to shunt use revealed that in studies reporting both selective and routine shunting an increased risk of stroke and death can be encountered in CO patients (resp., OR 1.83; 95% CI 1.34–2.52; *P* < 0.0002; OR 2.14; 95% CI 1.28–3.32; *P* < 0.0007), while in studies reporting no shunt use that risk was increased but not significantly (OR 2.61; 95% CI 0.91–7.46; *P* < 0.07). No CAS studies were deemed appropriate for the subgroup meta-analysis [35]. Nevertheless, based on historical data derived from RCTs, showing an increased risk of neurological complications in CO patients, a CAS treatment has been proposed and performed in the majority of those patients for many years. Keldahl et al. [36] retrospectively reviewed 417 CAS procedures performed between May 2001 and July 2010 and concluded that a preexisting CO does not seem to adversely impact CAS outcomes. On the contrary, a report by Brewster et al. [37] has pointed out that although CEA and CAS can both be performed with satisfactory perioperative results, the observed outcomes do not support the presence of contralateral carotid occlusion as a selection criteria for CAS over CEA in the absence of other indications. More recently, more papers have fuelled the debate on CO patients [38–40]. Yang et al. [38] have found that CO has no adverse impact on the development of postprocedural stroke after either CEA or CAS, reporting a stroke rate of 2.3% in 698 CEA patients routinely submitted to shunt implantation and 4% in 455 CAS patients. Ricotta et al. [39] have derived quite different conclusions, showing that in CEA patients CO significantly affects perioperative stroke rates (3.1% versus 1.1% in patients without CO, *P* < 0.0001), while in CAS patients CO does not affect periprocedural stroke rates (2.1% versus 2.3% in patients without CO, *P* = 0.82). Kang et al. [40] reported again CO to be among predictors of any stroke at 30 days after CEA and also of long-term ipsilateral stroke (HR 2.06; *P* = 0.025).

On the other hand, alternative strategies, such as medical therapy alone, appeared to be of low efficacy in patients affected by CO in historical studies [15, 41]. In those papers reporting on patients suffering from internal carotid artery stenosis and contralateral occlusion alarmingly high rates of recurrent stroke, ranging from 20% within 3 years to 34% within 51 months of follow-up in medically-treated patients, are recorded [41]. Data from NASCET [15] reported a 14.3% risk of stroke in CO patients submitted to CEA, but a 69.4% 2-year risk of stroke in those patients treated by medical therapy alone, thus leaving very short room to the latter, when considering the whole population of patients affected by CO. It must be acknowledged that optimal medical treatment has significantly improved since those cited studies were conducted, making it possible that medical therapy alone in asymptomatic patients with CO, as well as the so-called “high-risk” categories, should be considered the best treatment in the near future [42]. Hence, it is of outmost importance to identify those CO patients with additional risk factors for carotid surgery who can better be treated by optimized medical therapy and strict surveillance in absence of recent neurological symptoms.

## 5. Conclusions

In our experience CEA patients with CO present with a heavier burden of diffuse atherosclerotic disease when compared to control sample of CEA patients.

Moreover, they are at higher risk for postoperative neurological complications because of a higher brain susceptibility mainly encountered in a small subset of patients presenting association of two most significant risk factors for developing complications (advanced age, i.e., >74 years, and preoperative brain infarction on preoperative CT scans). In those cases the risk of surgery seems to be excessive, and exclusion from surgery with an alternative interventional or conservative approach could be worthwhile. Future studies evaluating the role of medical therapy over carotid intervention in this subgroup of patients are mandatory.

## Figures and Tables

**Table 1:** Analysis of demographic factors in 1639 primary carotid revascularizations.

	CO 136 cases		Control group 1503 cases		*P*
Age (years)	67.02 ± 7.9		69.72 ± 8.13		**<0.0001** (**95**% **CI**: **4.12 ± 1.27**)
Sex: males	114	83.8%	1054	68.3%	**0.001**
*Risk factors *					
Smoke	103	75.7%	828	53.7%	**<0.0001**
Hypertension	104	76.5%	1238	80.2%	0.111
Diabetes	42	30.9%	422	27.3%	0.551
Hyperlipidemia	60	44.1%	637	41.3%	0.763
Ischemic heart disease	41	30.1%	518	33.6%	0.356
Peripheral arterial disease	45	33.1%	248	16.1%	**<0.0001**
Abdominal aortic aneurysm	3	2.2%	72	4.7%	0.243
*Neurological presentation *					
Asymptomatic	50	36.8%	838	55.8%	**<0.0001**
Symptomatic	86	63.2%	665	46.4%	
TIA	60	44.1%	491	32.7%	
STROKE	26	19.1%	174	11.6%	
Shunt implantation	40	29.4%	121	8%	**<0.0001**
Infarct on neuroimaging^*^					**<0.0001**
Yes	65	47.8%	379	25.2%	
Ipsilateral to CEA	12	8.8%	213	14.2%	
Contralateral to CEA	41	30.2%	64	4.2%	
Bilateral	12	8.8%	102	6.8%	
No/NK	71	52.2%	1124	74.8%	

CO: contralateral carotid occlusion; NK: not known; ^*^detected by either brain CT or MRI.

**Table 2:** Perioperative (30-day) neurological outcomes in 1639 primary carotid revascularizations.

	CO 136 cases		Control group 1503 cases		*P*
Neurological deaths	2	1.5%	6	0.4%	0.283
Major stroke	6	4.4%	18	1.2%	**0.009**
Minor stroke	1	0.7%	8	0.5%	0.765

Overall neurological morbidity^*^	7	5.1%	26	1.7%	**0.016**
Overall neurological complications-combined endpoint°	9	6.6%	32	2.1%	**0.003**

CO: contralateral carotid occlusion; ^*^major + minor strokes; °neurological death + major strokes + minor strokes.

**Table 3:** Multivariate analysis of the role side of brain infarct related to the operated carotid artery on perioperative neurological outcome (stroke and stroke-related mortality) in 1639 primary carotid revascularizations (overall population brain lesion analysis OR 0.86, 95% CI 0.42–1.77, *P* = 0.69).

	CO group			Control group		
	OR	95% CI	*P*	OR	95% CI	*P*
Brain lesion overall	**4.47**	1.81–11.04	**0.0003**	0.56	0.23–1.34	0.19
Ipsilateral brain lesion	3.6	0.45–28.61	0.19	0.33	0.08–1.4	0.11
Contralateral brain lesion	**3.24**	0.95–10.96	**0.04**	0.6	0.08–4.5	0.62
Bilateral brain lesion	**8.14**	1.72–38.41	**0.001**	0.37	0.05–2.72	0.30

CO: contralateral carotid occlusion.

**Table 4:** Multivariate analysis of the role of age >74 years and brain lesion by side in 1639 primary carotid revascularizations.

	OR	95% CI	*P*
CO + >74 years	**11.54**	**4.08–32.62**	**<0.0001**
Control group + >74 years	0.57	0.25–1.29	0.17
CO + >74 years + contralateral brain lesion	**40.92**	**5.61–298.05**	**<0.0001**
CO + >74 years + ipsi- or bilateral brain lesion	**19.95**	**1.77–224.56**	**0.0006**
CO + >74 years + brain lesion overall	**28.68**	**7.76–105.93**	**<0.0001**

CO: contralateral carotid occlusion.
